# Critical Care Ultrasound in Shock: A Comprehensive Review of Ultrasound Protocol for Hemodynamic Assessment in the Intensive Care Unit

**DOI:** 10.3390/jcm13185344

**Published:** 2024-09-10

**Authors:** Camilo Pérez, Diana Diaz-Caicedo, David Fernando Almanza Hernández, Lorena Moreno-Araque, Andrés Felipe Yepes, Jorge Armando Carrizosa Gonzalez

**Affiliations:** 1 Critical and Intensive Care Medicine Department, Hospital Universitario Fundación Santa Fe de Bogotá, Bogotá 110111, Colombia; 2 School of Medicine and Health Sciences, Universidad del Rosario, Bogotá 111711, Colombia

**Keywords:** cardiac output, fluid responsiveness, hemodynamic monitoring, hemodynamic shock, point-of-care ultrasound, ventriculo-arterial coupling

## Abstract

Shock is a life-threatening condition that requires prompt recognition and treatment to prevent organ failure. In the intensive care unit, shock is a common presentation, and its management is challenging. Critical care ultrasound has emerged as a reliable and reproducible tool in diagnosing and classifying shock. This comprehensive review proposes an ultrasound-based protocol for the hemodynamic assessment of shock to guide its management in the ICU. The protocol classifies shock as either low or high cardiac index and differentiates obstructive, hypovolemic, cardiogenic, and distributive etiologies. In distributive shock, the protocol proposes a hemodynamic-based approach that considers the presence of dynamic obstruction, fluid responsiveness, fluid tolerance, and ventriculo-arterial coupling. The protocol gives value to quantitative measures based on critical care ultrasound to guide hemodynamic management. Using critical care ultrasound for a comprehensive hemodynamic assessment can help clinicians diagnose the etiology of shock and define the appropriate treatment while monitoring the response. The protocol’s use in the ICU can facilitate prompt recognition, diagnosis, and management of shock, ultimately improving patient outcomes.

## 1. Introduction

Shock is a common problem in the intensive care unit (ICU) with high mortality rates [1]. Early diagnosis is crucial to correct the cause, restore perfusion, and prevent organ failure [2]. Diagnosis is based on physical findings and biochemical signs of systemic hypoperfusion, but has limited value in differentiating the type and etiology, and assessing treatment response [3,4].

Invasive hemodynamic monitoring is a crucial strategy for resuscitation goals but has risks and is not immediately available [5]. Critical care ultrasound (CCU) is a quick, safe, and reliable bedside tool that provides new information in acute life-threatening conditions, changing clinical decisions in some cases. CCU improves critical care practice by characterizing shock type, guiding treatment initiation, and evaluating response [6,7].

A physiology-based approach to the patient admitted to the ICU for shock is necessary to guide resuscitation and hemodynamic support. This review aims to propose an ultrasound-based protocol for the hemodynamic assessment of the patient in shock using quantitative and qualitative measurements.

## 2. Hemodynamic Profiles

The traditional approach to shock classification is based on four hemodynamic parameters: cardiac index (CI), systemic vascular resistance (SVR), central venous pressure (CVP), and pulmonary artery occlusion pressure (PAOP). This approach defines four subtypes with different pathophysiological mechanisms: cardiogenic, hypovolemic, obstructive, and distributive (Table 1).

**Figure 1:**
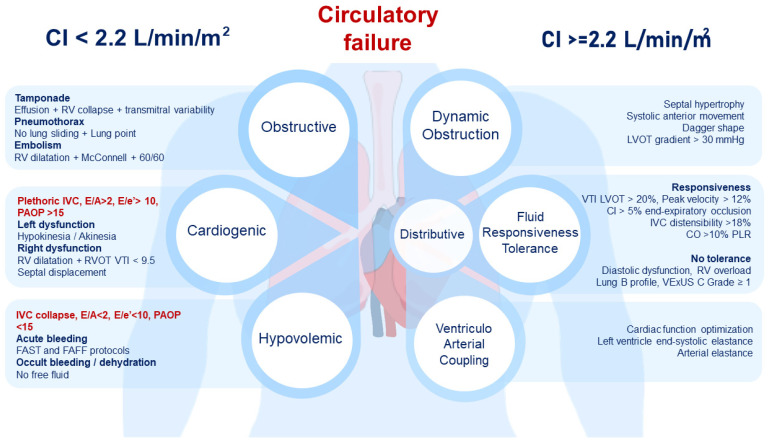
Hemodynamic profile of acute circulatory failure based on ultrasound. CI: cardiac index; CO: cardiac output; IVC: inferior vena cava; LVOT: left ventricle outflow tract; PAOP: pulmonary artery occlusion pressure; PLR: passive leg raising; RV: right ventricle; RVOT: right ventricle outflow tract; VTI: velocity–time integral.

CCU allows the classification of shock subtypes based on hemodynamic parameters estimated noninvasively. Ultrasound hemodynamic assessment begins with measuring CI using a formula that includes left ventricle outflow tract (LVOT) (Figure S1), velocity time integral (VTI) (Figure S2), heart rate, and the body surface area [8]. This approach classifies shock as low CI (≤2.2 L min^−1^ m^−2^) or high CI (>2.2 L min^−1^ m^−2^) [9,10]. The hemodynamic profile can be further classified based on a systematically ordered questions (Figure 1).

## 3. Low Cardiac Index Shock

Shock is diagnosed clinically, and its ultrasound-based approach can be ordered based on clinical findings. However, ruling out obstructive shock initially is recommended as it requires immediate interventions to resolve the shock.

### 3.1. Obstructive Shock

**Figure 2:**
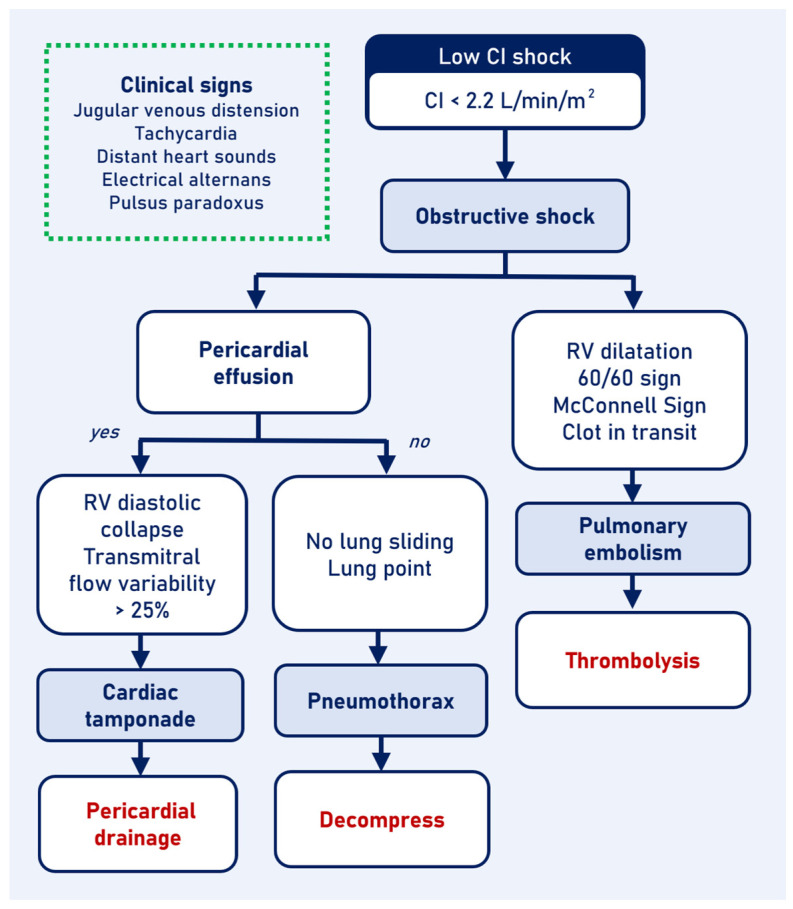
Low cardiac index shock algorithm. Obstructive shock causes. CI: cardiac index; RV: right ventricle.

Obstructive shock results from the collapse of great vessels or cardiac circulation. Clinical signs that suggest obstructive etiologies include jugular venous distension, tachycardia, distant heart sounds, electrical alternans, and pulsus paradoxus. Extravascular causes reduce diastolic filling, lowering right-side cardiac preload; these include tension pneumothorax, cardiac tamponade, and high positive end-expiratory pressure ventilation. Intravascular causes such as pulmonary embolism (PE) and pulmonary hypertension crises rapidly increase right ventricle (RV) afterload, quickly reducing stroke volume (SV) and left ventricular filling [11] (Figure 2).

#### 3.1.1. Identifying Pulmonary Embolism

A massive PE increases RV afterload, causing RV dilation and dysfunction [12]. In normal adults, the RV is smaller than the left ventricle (LV) and adapted to work in a low-pressure, low-resistance circulation. However, a sudden or chronic increase in pulmonary vascular resistance (PVR) can cause RV failure, resulting in chamber dilatation, decreased contractility, and reduced LV filling due to left septal displacement. This leads to a significant decrease in cardiac output (CO) [12,13]. Acute cor pulmonale—a consequence of severe RV overload—is defined based on RV dilatation with an RV/LV ratio greater than 1, flattening of the ventricular septum forming a D-shaped left cavity, and paradoxical septal motion at end-systole [12]. As RV pressures increase, the tricuspid annular systolic excursion (TAPSE) decreases to less than 16 mm, the venous return gradient decreases, and the inferior vena cava (IVC) dilates without inspiratory collapse [14,15]. RV systolic dysfunction can also be assessed through tissue Doppler imaging with a decreased peak systolic velocity of the tricuspid annulus (s′) less than 11 cm/s or a tricuspid regurgitation velocity (TRV) greater than 3.4 m s^−1^ [13,16,17] (Figure S5).

Specific findings of PE include a pulmonary acceleration time of less than ms with a peak systolic pressure gradient of less than 60 mmHg in the tricuspid valve (60–60 sign), decreased contractility of the free wall of the RV compared to the apex (McConnell’s sign), and the potential presence of a thrombus within the right cavities [18]. While most of these signs have low sensitivity if taken as isolated findings, when found together, they increase the positive predictive value (PPV). In patients with shock and suspected PE, the absence of echocardiographic signs of ventricular overload or dysfunction has the predictive power of excluding embolism as a cause of instability. The combination of these signs, especially McConnell’s sign, the 60–60 sign, or the presence of intracavitary thrombus within the right heart associated with hemodynamic instability, without other possible causes of decompensation in a patient with high clinical suspicion, justifies reperfusion therapy even in patients in whom it is not possible to perform other diagnostic methods, such as computed tomographic pulmonary angiography. However, long-standing pulmonary hypertension can cause RV hypertrophy with an RV free wall thickness greater than 4 mm. In this case, chronic pulmonary hypertension with cor pulmonale should be considered as a differential diagnosis and additional diagnostic tests should be performed [18].

#### 3.1.2. Identifying Pericardial Effusion

Pericardial effusion is frequently observed in the ICU, but its clinical significance lies in its impact on cardiac transmural pressures [19]. The detection of effusion depends on its size and distribution across various cardiac windows. The long parasternal axis can detect small posterior effusions, the apical four-chamber view can evaluate the movement of the atrial free wall, and the subcostal window is highly useful in assessing the effusion’s size, pericardial sac, and compression of cardiac chambers [20] (Figure S6).

Clinical diagnosis of CT is supported by CCU [21]. Characteristic signs include respiratory variation in the size of right cavities, collapse of atrial cavities during systole, collapse of the RV free wall during diastole, and a plethoric IVC without respiratory variations [22]. Systolic collapse of the RA is one of the earliest signs of cardiac tamponade, with a sensitivity of 50% in the early phase and 100% in the late phase [21]. In patients with increased right ventricular filling pressures due to pulmonary hypertension, hypervolemia, or RV hypertrophy, the transmural pressures necessary for cavity collapse may be higher, leading to a loss of compression effect. Hypovolemia may magnify the effect, particularly atrial compression, without necessarily indicating a true cardiac tamponade. Therefore, Doppler examination should be performed to confirm the diagnosis of cardiac tamponade [23].

When hemodynamic repercussions are present, the respiratory variations of systolic volume increase due to ventricular interdependence caused by pericardial effusion. This is manifested in spontaneously breathing patients by a reduction in the peak velocity of the mitral E wave during inspiration greater than 25% and a transtricuspid flow greater than 40% [24].

#### 3.1.3. Identifying Pneumothorax

In the assessment of obstructive shock, ruling out tension pneumothorax is essential. CCU has high sensitivity (86–98%) and specificity (97–100%) for pneumothorax diagnosis [25]. Pneumothorax separates the visceral and parietal pleura, preventing visualization of the visceral pleura and resulting in an absence of lung sliding. M-mode exploration can reveal the stratosphere sign, characterized by horizontal lines above and below the pleura due to the absence of movement [26].

The presence of lung sliding has a high negative predictive value (NPV) (99–100%) for diagnosing pneumothorax [27]. The presence of B-lines or artifacts in comet tails also has a high NPV and can rule out pneumothorax if observed [28]. However, the absence of lung sliding does not always indicate the presence of pneumothorax, as other differential diagnoses include selective intubation, consolidations, and diaphragmatic paralysis [29]. The pulmonary point is a highly specific sign for pneumothorax, characterized by lung sliding in some sections of the pleura while being absent in others where the pneumothorax begins [30] (Figure S8).

### 3.2. Cardiogenic Shock

**Figure 3:**
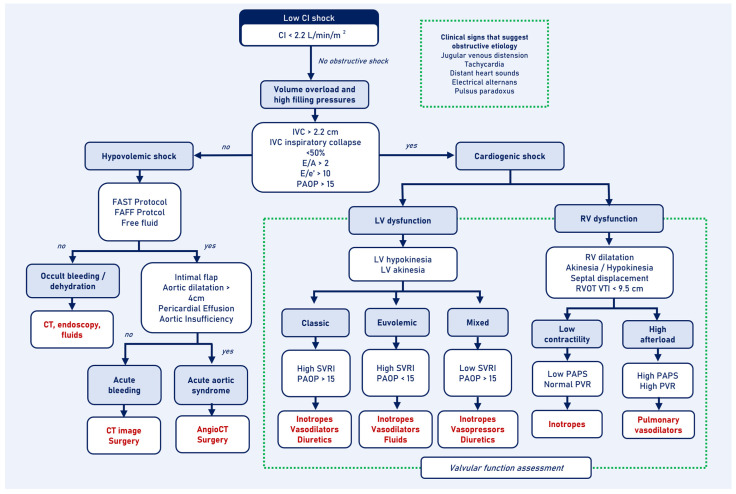
Low cardiac index shock algorithm: cardiogenic and hypovolemic shock. CI: cardiac index; CT: computed tomography; IVC: inferior vena cava; LV: left ventricle; PAOP: pulmonary artery occlusion pressure; PAPS: pulmonary artery systolic pressure; PVR: pulmonary vascular resistance; RV: right ventricle; RVOT: right ventricle outflow tract; SVRI: systemic vascular resistance index; VTI: velocity–time integral.

Once obstructive etiologies are ruled out, the assessment of patients with low CI shock should focus on distinguishing between cardiogenic and hypovolemic shock, based on volume and filling pressures [31]. CCU is a highly effective tool for differentiating between the two types of shock. Qualitative assessment protocols have a PPV of 91.7% and NPV of 97% for diagnosing cardiogenic shock [32]. However, using quantitative measurements increases the NPV to 100% with high interobserver agreement [32,33] (Figure 3).

#### 3.2.1. Evaluating for Increased Filling Pressures and Hypervolemia

IVC diameter and inspiratory collapse serve as noninvasive surrogates for right atrial pressure (RAP), which reflects the relationship between venous return and cardiac function [34]. A diameter greater than 2.1 cm and inspiratory collapse less than 50% suggest a RAP of at least 15 mmHg [35]. However, various factors, such as measurement site, inspiratory effort, weight, intra-abdominal pressure, intrathoracic pressures, and mechanical ventilation, moderate the correlation between the noninvasive estimate and the invasive measurement [36,37]. Hence, isolated use of IVC diameter and inspiratory collapse has a low diagnostic performance [38,39].

Diastolic function and filling pressures have a close association [40]. CCU allows assessment of transmitral blood flow to identify the E and A waves and the longitudinal elongation of fibers at the mitral annulus level during early diastole using tissue Doppler to identify the e′ wave, which reflects LV relaxation rate independent of filling conditions [41,42]. These waves enable the estimation of pressure gradients during diastole, from which the filling pressure of the left heart may be inferred. The E/e′ ratio correlates with the PAOP across a wide range of clinical conditions, and it is possible to estimate the mean value of PAOP (PAOP = 1.91 + (1.24 × E/e′)) with low intra- and interobserver variability [42]. Therefore, an E/A wave ratio greater than 2, an E/e′ ratio greater than 10, or an estimated PAOP greater than or equal to 15 mmHg indicate increased filling pressures [40,43].

Organ congestion is the ultimate consequence of high filling pressures. CCU enables the identification of lung congestion by the presence of a diffuse B profile and intra-abdominal congestion using the VExUS-C protocol [39].

#### 3.2.2. Assessing for Signs of Left Ventricular Dysfunction

LV dysfunction assessment starts with identifying global or segmental contractility disorders using qualitative methods based on wall thickening, endocardial motion, and end-systolic volume estimation. Qualitative protocols have demonstrated high throughput and reproducibility in diagnosing cardiogenic shock [44]. LV dysfunction should be considered when a low CI is present, along with signs of congestion or increased filling pressures and a hypokinetic or akinetic heart.

CCU allows for the hemodynamic characterization of cardiogenic shock with left ventricular dysfunction using CI, PAOP, and estimated systemic vascular resistance index (SVRI). There is a high correlation (r = 0.86) between SVRI measured by transpulmonary thermodilution and SVRI estimated by CCU using the SRVI formula (SRVI dynes.s. m^2^/cm^−5^) = (MAP − RAP) (mmHg) × 80/CI (L min^−1^ m^−2^) [45]. This approach classifies shock into three profiles: classic shock with low CI, high SRVI, and high PAOP, which benefit from inotropes, vasodilators, and diuretics; euvolemic shock with low CI, high SRVI, and normal or low PAOP, where intravenous fluids should be considered; and mixed shock with low CI, low SRVI, and high PAOP, where vasopressors are beneficial [46].

#### 3.2.3. Assessing for Signs of Right Ventricular Dysfunction

RV dysfunction is a frequent event in ICU patients. The abrupt increase in RV afterload triggers the mechanism for RV failure, especially when mechanically ventilated. RV failure follows a time sequence of events beginning with dilatation, paradoxical septal motion, and RV systolic dysfunction as a final step of RV and pulmonary artery uncoupling. RV function assessment involves evaluating morphology and contractility to identify dilated cavities, decreased contractility, and paradoxical septum movement. However, interobserver variability is high, and it is necessary to supplement qualitative assessment with quantitative measures [47].

The fractional area change (FAC) and TAPSE are standard measures to assess RV systolic function. FAC correlates with right ventricular ejection fraction (RVEF) assessed by cardiac MRI [48]. Values less than 35% are abnormal [49] and associated with worse outcomes in pulmonary hypertension, PE, and acute respiratory distress syndrome (ARDS) [50]. TAPSE represents RV longitudinal shortening during systole [51], and is reproducible and correlated with RVEF. Values less than 17 mm are abnormal [52] and predict mortality in critically ill patients (54), ARDS (55), and septic shock [53,54,55]. However, FAC and TAPSE do not consider the contribution of the right ventricular outflow tract (RVOT) to systolic function and do not accurately define the development of RV failure [56,57] (Figure S3).

Measuring the RVOT VTI allows for estimation of RV-SV if combined with RVOT diameter measurement (RV-SV = RVOT VTI × RVOT area). Due to difficulties in measuring RVOT diameter accurately, it is recommended to use VTI as a surrogate for RV-SV [8]. This method has an 88% reliability rate in critically ill patients [58]. A normal RVOT VTI value in healthy adults is 18 to 22 cm with a peak velocity of 0.8 to 1.2 L s^−1^ [59]. Low RVOT VTI values (<9.5 cm) are independent predictors of mortality in PE [58] (Figure S4).

RV failure occurs when RV systolic properties are unable to overcome afterload, resulting in a vicious circle of overload during systole reflected in paradoxical septum movement, overload during diastole with development of dilatation, low RV-SV, and LV diastolic dysfunction with signs of increased filling pressures [56]. Noninvasive estimation of pulmonary artery systolic pressure (PAPS) and PVR allows the identification of the predominant mechanism of RV failure. PAPS can be estimated by measuring the pressure gradient of the TRV if RAP is known using Bernoulli’s equation (PAPS = 4 × TRV^2^ + RAP) [60]. PAPS estimated by ultrasound correlates well with right-heart catheterization, even under mechanical ventilation, but limitations arise in an unfavorable acoustic window, measurements not parallel to the regurgitation jet, and imprecise estimates of the RAP [61].

To estimate PVR, if the mean pulmonary artery pressure (PAPm), PAOP, and CO are known, PVR can be calculated using the formula PVR = PAPm − PCWP/CO [62]. PAPm can be obtained using the formula of Chemla et al. (PAPm = PAPS × 0.61 + 2 mmHg), which shows a high correlation (r = 0.87) between CCU, right-heart catheterization, and high diagnostic performance in identifying patients with PVR > 3 UWoods [62,63]. However, it is a complex method, and other simpler estimates have been proposed. The morphology of the VTI RVOT can suggest the presence of increased PVR secondary to the reflection velocity of the pulse wave in the pulmonary circulation. The RVOT VTI acceleration time, measured from the start of pulmonary flow to peak systolic velocity, allows quantification of this phenomenon. Normal values are greater than 130 msec, and values less than 100 msec correlate with high PVR [59].

Patients with right ventricular failure and systolic dysfunction will have normal or low PAPS without increases in PVR, and they will benefit from inotropes. In cases where afterload predominates, PAPS will be elevated with increased PVR, and pulmonary vasodilators will be beneficial. However, scenarios may arise with mixed compromise, in which PAPS is decreased with increases in PVR, and these cases will benefit from both inotropes and pulmonary vasodilators.

#### 3.2.4. Assessing for Signs of Severe Valve Disease

Valvular function assessment is a complex process that requires advanced echocardiography training [64]. Nonetheless, qualitative assessment can identify significant valvular disorders that may cause shock. A systematic approach is necessary to assess obstructions and regurgitation by identifying valve thickening, calcification, vegetations, or immobility, complemented with color Doppler to identify turbulent flow and regurgitation magnitude [65]. Aortic stenosis screening should describe the degree of decrease in opening, the presence of calcification or sclerosis, and cusp mobility. Aortic insufficiency screening should identify the regurgitation jet distribution in the transverse and longitudinal planes along the LVOT. Mitral insufficiency screening should identify the extension of the regurgitation jet within the left atrium and its distribution. Mitral stenosis screening should assess the presence of calcifications, sclerosis, reduced cusp mobility, and paradoxical posterior movement during diastole [66].

CCU is essential for cardiogenic shock assessment. However, limitations exist, such as a poor acoustic window, biventricular dysfunction, severe valve disease, and refractory cardiogenic shock, requiring complementary hemodynamic monitoring with a pulmonary artery catheter (PAC) and transesophageal echocardiography.

### 3.3. Hypovolemic Shock

#### Identifying Low Intravascular Volume

Patients with low CI, low filling pressures (E/A 0.8–2 with E/E′ < 10), small chambers, hypercontractile heart, lung profile A, and a collapsed IVC (<2 cm with >50% variability with respiration) measured at two centimeters from the cavo-atrial junction likely present with hypovolemic shock [67,68,69].

Ultrasound assessment of hypovolemic shock assessment must differentiate between traumatic and nontraumatic shock. The FAST protocol has the best evidence for traumatic hemorrhagic shock, ruling out free intra-abdominal fluid, cardiac tamponade, pneumothorax, or hemothorax at the bedside in less than three minutes [70,71]. A prospective cohort study demonstrated sensitivities and specificities of 81.5% and 99.7% for general trauma, 78.6% and 100% for blunt injuries, and 83.8% and 97.4% for open trauma, respectively [71].

Pleural spaces can also be assessed to identify free fluid, with a sensitivity greater than 92% and specificity close to 100%. Hemothorax may be considered when the following criteria are met: the presence of an anechoic fluid with moving points and two liquid densities, movement of the atelectatic lung due to a pleural effusion (known as the jellyfish sign), visualization of the vertebral bodies in the thoracic cavity above the diaphragm (spine sign) (73), absence of dynamic movement of the pulmonary curtain, and no visualization of the lateral diaphragm (known as the curtain sign) [72,73].

In unstable patients, a positive test indicates the need for surgery, while a negative test requires another test such as angio-CT or peritoneal lavage. In hemodynamically stable patients, a positive test requires another confirmatory test such as angio-CT or, depending on the context of the surgical procedure, a negative test requires strict follow-up or an angio-CT [74]. However, the FAST protocol has limitations, including examiner dependence and injuries to the solid organs, intestine, mesentery, diaphragm, and retroperitoneum. It also has limitations in patients with obesity, subcutaneous emphysema, late presentation, ascites, fluid-filled intestine, renal cyst, or stones. A meta-analysis has shown a sensitivity of 74% and specificity of 98%, with high heterogeneity [75].

Measures to improve the accuracy of the FAST protocol include repeat examination during the first 24 h of ICU admission, contrast media use, the use of the R-E-FAST protocol to look for retroperitoneal hematomas, and the E-FAST + SLOOW protocol to assess free fluid between loops with a linear probe [76,77]. Additional findings to screen for include pneumoperitoneum, parenchymal heterogeneity in liver or spleen trauma, and double line sign between the hyperechoic capsule and the hypoechoic liquid in renal trauma. [78].

In nontraumatic hemorrhagic hypovolemic shock, the FAFF (Focused Assessment for Free Fluid) test [79] uses the same windows as the FAST protocol and is effective in cases of ruptured aneurysms, ruptured ectopic pregnancy, and sepsis. To rule out intra-abdominal bleeding caused by ruptured abdominal aneurysm, a complete longitudinal and transverse plane examination should be conducted, looking for dilations of more than 3 cm from the epigastrium to the iliac bifurcation, with a sensitivity of 93–100% and a specificity approaching 100% [80].

## 4. High Cardiac Index Shock

### 4.1. Distributive Shock

Distributive shock results from the regulation loss of peripheral vascular resistance [81]. However, myocardial dysfunction and hypovolemia coexist frequently and pose a challenge for comprehensive hemodynamic evaluation [82]. Consequently, CCU protocols have lower sensitivity, specificity, precision, and agreement for classifying distributive shock [32,33,82,83,84]. This lower performance may be due to protocols that rely on LVOT VTI measurements to categorize patients with low LVOT VTI values as having hypovolemic or cardiogenic shock, without considering the contribution of heart rate and LVOT diameter on the absolute CI value. Vignon et al. conducted a prospective study on patients with septic shock to assess the agreement in shock classification based on hemodynamic profile obtained by transpulmonary thermodilution and CCU. The average heart rate, cardiac index, and LVOT VTI were 110 beats/min, 3.25 L min^−1^ m^−2^, and 15 cm, respectively. Despite the low LVOT VIT, in this group of distributive shock patients, the cardiac index remained normal or high [82].

**Figure 4:**
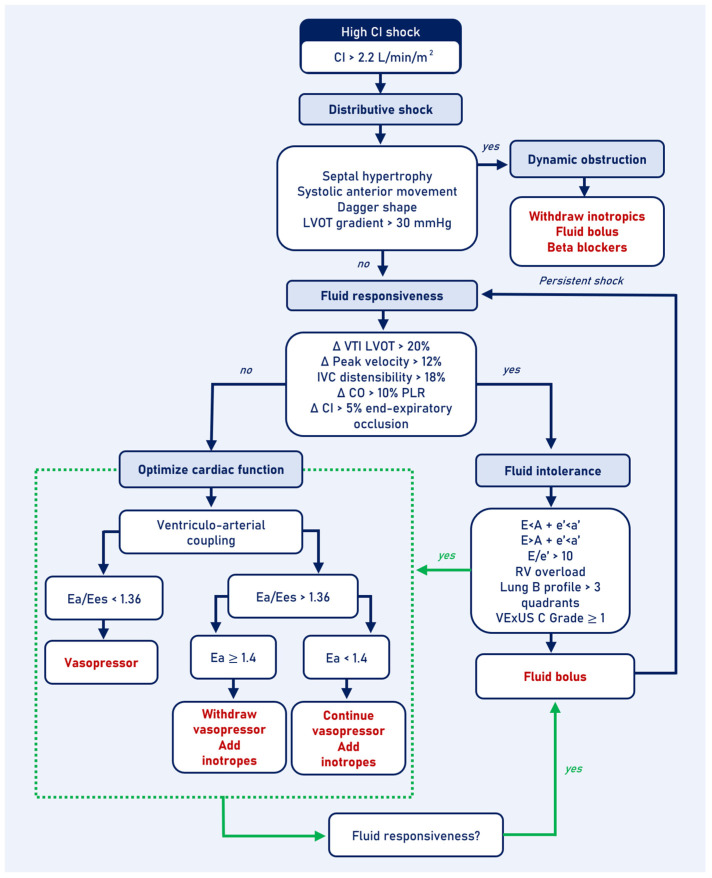
High cardiac index shock algorithm. CI: cardiac index; LVOT: left ventricle outflow tract; IVC: inferior vena cava; CO: cardiac output; PLR: passive leg raising; Ea: arterial elastance; Ees: left ventricular end-systolic elastance; RV: right ventricle.

The assessment of distributive shock begins with demonstrating a normal or elevated CI of >2.2 L min^−1^ m^−2^ [33,85]. The use of isolated LVOT VTI values is not recommended. In scenarios where measuring LVOT diameter is not possible, a VTI > 15 cm is suggested as a cutoff to identify a normal or high CI [82]. Hemodynamic profiles can vary by disease stage, determine the impact of interventions, and can be classified into three phenotypes: dynamic obstruction, fluid response, and ventriculo-arterial decoupling (Figure 4).

#### 4.1.1. Assessing for Dynamic Obstruction

Dynamic obstruction is a frequent and serious event in the ICU that is associated with a high mortality rate and cannot be accurately identified with PAC [86]. Obstruction can occur at the LVOT level due to systolic anterior motion (SAM) of the anterior mitral leaflet or at the intraventricular level due to hypovolemia, hyperkinesia, and decreased left ventricular afterload [87]. Obstructive flow patterns are present in 22% of patients with septic shock 6 h after admission to the ICU, with 36% at the midventricular level, 24% at the LVOT level, and 40% in both [86].

Dynamic obstruction can cause catecholamine-refractory distributive shock that worsens with increased inotropes [88]. CCU findings include a hyperkinetic pattern, decreased left ventricular end-diastolic area (<10 cm^2^), septal hypertrophy, turbulent flow in the LVOT, and SAM. However, the sign that defines the obstruction is the morphology of the continuous wave spectral Doppler with a high-velocity, dagger-shaped late systolic peak, and peak pressure gradient ≥ 30 mmHg [89].

Treatment involves correcting the triggering factors by increasing afterload and preload, decreasing heart rate, and reducing hyperkinesis [90]. Initially, it is suggested to suspend inotropes and diuretics, administer fluid boluses, and in refractory cases, consider starting selective β1 beta blockers [91].

#### 4.1.2. Prediction of Fluid Responsiveness and Tolerance

Assessing fluid response helps identify patients with a high probability of increased CO and oxygen delivery (DO2) after a fluid bolus. Fluid responsiveness is defined as a 10–15% increase in CI or SV following a fluid bolus [92]. At ICU admission, 50% of septic shock patients are responders, and the remaining 50% are nonresponders [93]. However, not all fluid responders require a fluid bolus, and some may only have transient increases in CI with the risk of congestion [94].

Fluid response tests are classified as static or dynamic. Dynamic tests are preferred due to their better predictive capacity [37,95]. Tests derived from pulse wave contour analysis, such as pulse pressure variation and systolic volume variation, require conditions that are met in only a limited number of patients in the ICU. Therefore, measurements obtained from ultrasound are valuable for predicting fluid responsiveness in these scenarios. Ultrasound-based measurements include LVOT peak systolic flow velocity variability > 12%, LVOT VTI variability > 20%, IVC distensibility > 18%, the passive leg raising (PLR) test with a 10% increase in CO, and the 15 s pause at end-expiration in patients with mechanical ventilation with a 5% increase in CI [96,97,98,99,100]. However, fluid administration decisions should not solely be based on the probability of increased CO after a bolus (fluid responder) but should also consider the cardiovascular system’s capacity to accept fluids without developing congestion (fluid tolerance). This relationship is determined by cardiac function, and therefore, tolerance will depend on the ability to maintain normal filling pressures after a fluid bolus [101].

A prospective study evaluated changes in diastolic function after a fluid bolus between responders and nonresponders, demonstrating that the increase in the E/e′ ratio occurred only in the subgroup of nonresponders with diastolic dysfunction, with no differences between responders and nonresponders without diastolic dysfunction and no correlation between ∆E/e′ and ∆SV [102]. Thus, four phenotypes of patients can be identified: tolerant responders who will benefit, nontolerant responders who will not benefit, tolerant nonresponders who will not benefit, and nontolerant nonresponders who will not benefit.

Characterizing venous congestion risk at the pulmonary, cardiac, great veins, hepatic circulation, portal circulation, and kidney level is essential in CCU [103]. At the cardiac level, it can be assessed by means of transmitral flow gradients with the presence of diastolic dysfunction with an E < A and e′ < a′ wave, E > A wave and e′ < a′, and an E/e′ wave > 10, suggesting high PAOP [104]. Signs of right ventricular (RV) overload such as a D-shaped septum, paradoxical septum movement, and RV/LV ratio > 1 represent a high risk of congestion [105]. At the pulmonary level, the presence of pleural effusion and B profile in at least three of eight pulmonary quadrants have been correlated with increases in extravascular lung water index (EVLWi) > 10 mL kg^−1^ [106]. Congestion in large veins can be assessed using the VExUS-C score, with a grade ≥ 1 considered high risk of congestion associated with hemodynamic compromise on renal perfusion [107].

In distributive shock patients with evidence of preload dependence indicating a high likelihood of response to fluids, and without signs of intolerance suggesting low risk of congestion, administration of a crystalloid bolus of at least 4 mL kg^−1^ is recommended [94]. Response should be assessed. If there is evidence of preload dependency, but a high risk of congestion is present or there is an absence of preload dependency, ventriculo-arterial coupling should be assessed to determine the best strategy for optimizing cardiac function, which may involve increasing venous return, shifting to the ascending portion of the Frank–Starling curve, or improving congestion before considering administering a fluid bolus.

#### 4.1.3. Assessing for Ventriculo-Arterial Decoupling

Clinical grounds for the decision to withdraw vasopressors or introduce inotropes are mostly related to the value and stability of mean arterial blood pressure, LV systolic function, stroke volume, and signs of tissue hypoperfusion. An approach based on ventriculo-arterial coupling allows the interpretation of the contribution of ventricular function and vascular function to SV for a set value of preload and venous return, to guide the choice between fluids, vasopressors, and inotropes [83,108].

Ventriculo-arterial coupling is useful for interpreting the mechanical efficiency of the cardiovascular system based on the relationship (Ea/Ees) between arterial elastance (Ea) and left ventricular end-systolic elastance (Ees) [109], a parameter that reflects contractility and is not dependent on filling conditions [110]. Elastance represents the pressure changes necessary to generate a volume change, which are a function of time throughout the cardiac cycle and represent the magnitude of energy needed to generate the ejection and flow of a given SV [111] (Figure S7).

To improve DO2 efficiently, a point of maximum conservation of energy in the cardiovascular system must be achieved [112]. Maximum mechanical efficiency is reached with a ratio of Ea/Ees = 0.8 ± 0.16 [113]. However, in normal adults, the ratio of Ea/Ees = 1 ± 0.36 is assumed as the equilibrium point, with normal Ea values of 2.2 ± 0.8 mmHg mL^−1^ and 2.3 ± 1 mmHg mL^−1^ for Ees [109].

Ventriculo-arterial decoupling, defined as an Ea/Ees ratio > 1.36, is frequent in septic shock. Although both Ea and Ees decrease, the reduction in Ees is greater and this explains the higher values of the Ea/Ees ratio [114]. Ventriculo-arterial decoupling is associated with worse tissue perfusion and a higher risk of mortality in septic shock [115]. Resuscitation strategies that optimize the Ea/Ees ratio close to 1 have been associated with improved perfusion and a trend towards lower mortality at 28 days (hazard ratio 0.5; 95% CI 0.258–1.033) [83,108].

An ultrasound method for calculating ventriculo-arterial coupling based on five parameters has been validated [114,116]. These parameters are systolic blood pressure, diastolic blood pressure, SV, left ventricular ejection fraction estimated by the Simpson method, and normalized ejection time estimated from the total ejection time and the pre-ejection time in the Doppler spectral flow in the LVOT [117]. The description of the mathematical principles that dictate the relationship between these variables is beyond the scope of this article. However, there are applications (iElastance^®^ [118], Oxybol^®^) available to perform the calculation at the patient’s bedside.

In cases of distributive shock without dynamic obstruction or preload dependency, it is suggested to assess the Ea/Ees ratio. An Ea/Ees ratio of less than 1.36 represents low Ea with preserved ventricular contractility, and it is suggested to continue with vasopressor support. However, an Ea/Ees ratio greater than 1.36 could be explained by low values of both Ea and Ees but with a greater reduction in Ees. Therefore, it is necessary to evaluate the absolute values of Ea and Ees. If Ees is less than 1.3 and Ea is greater than or equal to 1.4, it is suggested to increase inotropic support and decrease the vasopressor dose until Ea is normalized. If Ees is less than 1.3 and Ea is less than 1.4, it is suggested to continue with the vasopressor and add inotropes [83].

In the case of distributive shock without dynamic obstruction but with preload dependence and poor tolerance to fluids, it is suggested to optimize ventriculo-arterial coupling before fluid administration. If there is persistent high Ea/Ees despite high doses of vasopressor or inotropic support, it is suggested to administer a crystalloid bolus of at least 4 mg kg^−1^ and reassess the response [119].

## 5. Conclusions

CCU has emerged as a valuable tool for the hemodynamic assessment of shock in the ICU, providing clinicians with a reliable and reproducible tool to diagnose and classify this life-threatening condition. The proposed ultrasound-based protocol for shock management presents a hemodynamic-based approach that considers both etiology and quantitative measures to guide appropriate treatment and monitoring. Its implementation can lead to improved patient outcomes by facilitating early recognition, timely intervention, and tailored management.

## Figures and Tables

**Table 1:** Hemodynamic profiles of shock. CI: cardiac index; CVP: central venous pressure; PAOP: pulmonary artery occlusion pressure; SVR: systemic vascular resistance.

Shock Subtype	CI	SVR	CVP	PAOP
Cardiogenic	Low	High	High	High
Hypovolemic	Low	High	Low	Low
Obstructive	Low	High	High	High
Distributive	High	Low	Low	Low

## Data Availability

No new data were created or analyzed in this study. Data sharing is not applicable to this article.

## Supplementary Materials

The following supporting information can be downloaded at: https://www.mdpi.com/article/10.3390/jcm13185344/s1, Figure S1: Left ventricular outflow tract diameter; Figure S2: Left ventricular outflow tract velocity time integral; Figure S3: Tricuspid annular plane systolic excursion; Figure S4: Right ventricular outflow tract velocity time integral; Figure S5: Ultrasound signs in acute pulmonary embolism; Figure S6: Pericardial Effusion; Figure S7: Pre-ejection and total ejection time; Figure S8: Lung ultrasound signs.
