# Supplementary figures and images for: Critical Care Ultrasound in Shock: A Comprehensive Review of Ultrasound Protocol for Hemodynamic Assessment in the Intensive Care Unit
Source: J Clin Med. 2024 Sep 10;13(18):5344. doi: 10.3390/jcm13185344 (PMC11432640; doi:10.3390/jcm13185344)

Figure S1

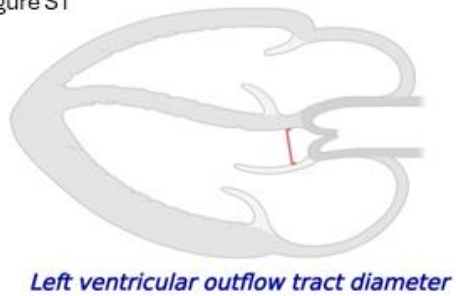

Figure S2

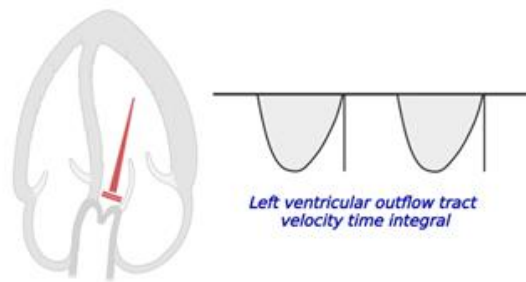

Figure S3

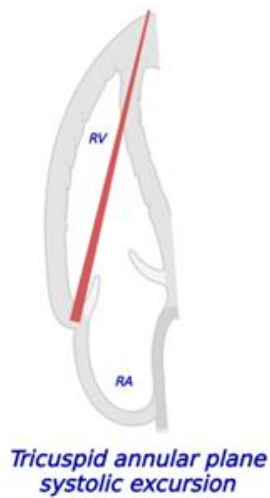

Figure S4

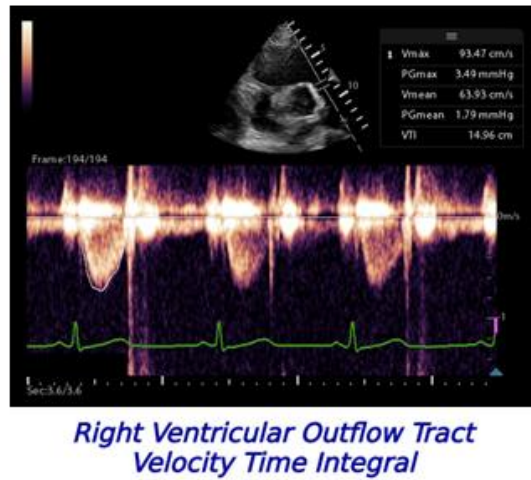

Figure S5

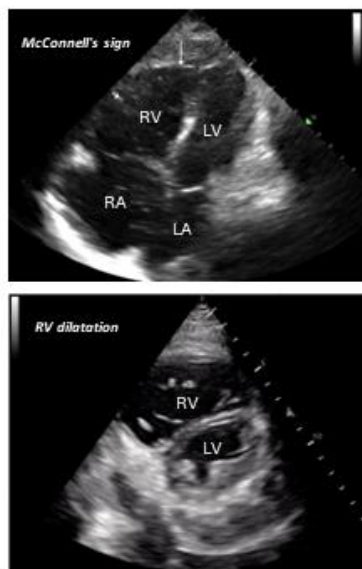

Figure S6

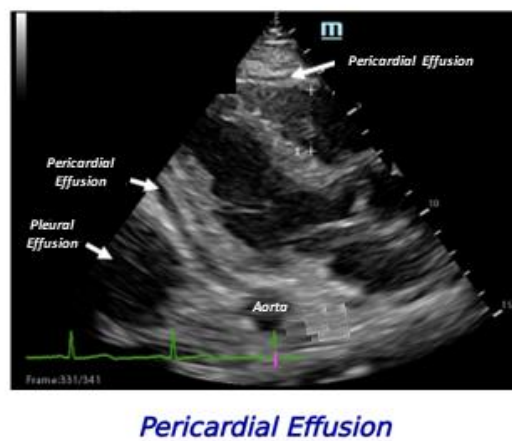

Figure S7

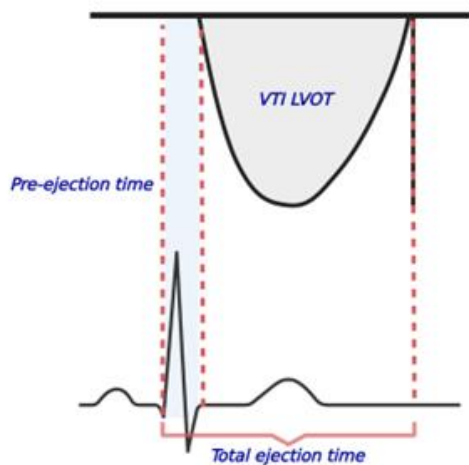

Figure S8

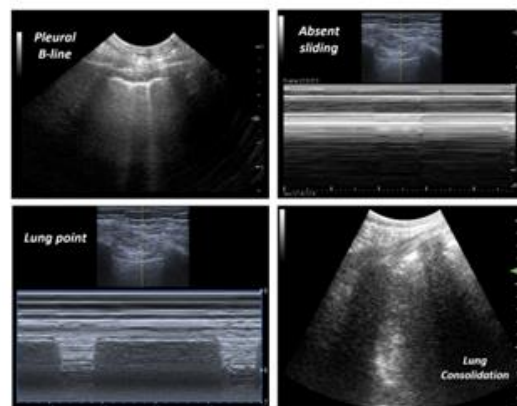

Supplement: Supplementary file 1 [file jcm-13-05344-s001.zip › jcm-3084756-supplementary.pdf]
